# Wasteful carotenoid coloration and its effects on territorial behavior in a cichlid fish

**DOI:** 10.1007/s10750-020-04354-3

**Published:** 2020-08-18

**Authors:** Angelika Ziegelbecker, Klaus Remele, Hartwig W. Pfeifhofer, Kristina M. Sefc

**Affiliations:** 1 Institute of Biology, University of Graz, Universitätsplatz 2, 8010 Graz, Austria; 2 Institute of Biology, University of Graz, Schubertstraße 51, 8010 Graz, Austria

**Keywords:** *Tropheus*, Agonistic behavior, Color pattern, Intimidation, Signaling, Integumentary carotenoid concentration

## Abstract

The famously diverse body coloration of cichlid fish serves communicative functions in mating and social interactions including competition for resources. Here, we examined the effects of a color pattern trait—the width of a yellow bar on a black body—on territorial competition in males and females of a color variant (“Ikola”) of the Lake Tanganyika cichlid *Tropheus*. First, measuring integumentary carotenoid concentrations in the yellow and black body regions, we established that wider yellow bars require more carotenoids allocated to body coloration. However, we also detected high carotenoid concentrations in the black body regions (> 100 µg/g fresh skin), raising questions about the function of non-displayed integumentary carotenoids. Behavioral experiments showed that fish with wider bars were quicker to explore an unfamiliar area of the tank. In experiments including presentations of fish dummies, the bar width of ‘territorial’ dummies had no effect on the latency time which test fish took to intrude into the dummies’ territories. However, male test fish performed fewer aggressive acts against wide-barred than against narrow-barred dummy competitors. Our results suggest that intimidation by wide bars as well as correlations between bar width and explorative behavior may contribute to mediating success in territorial *Tropheus* “Ikola”.

**Electronic supplementary material:**

The online version of this article (10.1007/s10750-020-04354-3) contains supplementary material, which is available to authorized users.

## Introduction

**Fig. 1:**
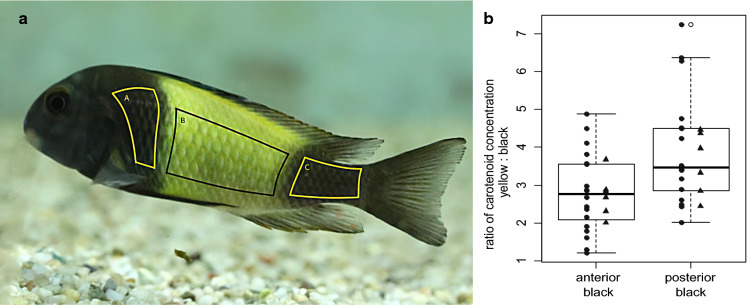
A photograph of *Tropheus* sp. ‘black’ from Ikola, Tanzania (**a**). Regions of the integument that were examined for carotenoid concentrations are indicated (A, black area anterior to the yellow bar; B, yellow bar region; C, black area posterior to the yellow bar). The photograph was taken by AZ in the fish facility of University of Graz. **b** Carotenoid concentration (measured as µg/cm^2^ body surface) in the yellow relative to the anterior and posterior black body regions. The boxplots show the distribution of datapoints pooled across sexes; filled circles and triangles represent datapoints from individual males (*n* = 18) and females (*n* = 6), respectively

Color signals are an important means of communication across the animal kingdom, functioning in various contexts ranging from mate choice and competition to predation avoidance (Maan & Sefc, 2013; Cuthill et al., 2017). In contest situations, body coloration can convey information about status and fighting ability and hence be used to assess opponents and evade unwinnable conflicts, reduce escalation and avoid injury (Rohwer, 1975; Maynard Smith & Harper, 2003; Senar, 2006; Blount & McGraw, 2008). Certain color patterns, notably those including red coloration, have been shown to be associated with dominance in a way that is sensitive to color masking and manipulation (Evans & Norris, 1996; Baube, 1997; Dijkstra et al., 2005; Healey et al., 2007) and likely based on the intimidation of opponents (Pryke, 2009). Additionally, body coloration can correlate with behavioral and physiological traits that are of advantage in contest situations, such as aggressiveness, boldness, endurance or overall levels of activity (cichlids: Schweitzer et al., 2015; Balzarini et al., 2017; Hermannn’s tortoises: Mafli et al., 2011; side-blotched lizards: Sinervo et al., 2000; Arctic char: Backström et al., 2014). Several studies demonstrated that not only the hue but also the size of colored areas can be correlated with competitive success and dominance (Viera et al., 2008; Järvistö et al., 2013; Dey et al., 2017; Tinghitella et al., 2018). This was also the case in the cichlid fish *Tropheus* sp. ‘black’ (sensu Konings, 2019), where the population from Ikola (Lake Tanganyika, Tanzania) displays a wide yellow bar on a black body (Fig. 1a, from here on referred to as *Tropheus* “Ikola”). In a recent study, we found a correlation between the outcome of contest competition and the width of the yellow bar, as winners of experimentally staged female–female contests for territories had wider bars than their opponents on average (Ziegelbecker et al., 2018). In contrast, no evidence for a connection between bar width and contest outcome was found in male–male contests, although *Tropheus* “Ikola” are sexually monomorphic in color pattern, body size and territorial behavior (Ziegelbecker et al., 2018). In their natural environment, males and females defend their individual feeding territories in the shallow rocky littoral habitat of Lake Tanganyika, East Africa. Contestants employ rapid physiological color changes for communication during agonistic interactions (Yanagisawa & Nishida, 1991; Sturmbauer & Dallinger, 1995). To signal subordination, *Tropheus* “Ikola” subdue the intensity and contrast of their color pattern by dispersing the pigment in the melanophores of the bar region and aggregating pigment in the melanophores of the black region. This results in pale, greyish body coloration, whereas dominant individuals display a strong contrast of intense yellow and dark black coloration. Contrary to this flexibility in the display of color intensity, the width of the yellow bar (in relation to body size) remains constant for months and years after the formation of the adult color pattern (Ziegelbecker et al., 2018). Based on the role of physiological modifications of the color pattern during agonistic interactions, we suggested in our previous study that the competitive advantage of wide-barred females may reflect a signaling function of the morphological variation in bar width.

The yellow coloration of the bar is produced by carotenoids (Ziegelbecker et al., 2018). Animals acquire carotenoids through their diet and metabolize them to other carotenoid compounds or derivatives with crucial functions in physiological processes that can compete with their allocation to body coloration (Svensson & Wong, 2011). The intensity of carotenoid-based coloration of animals is often condition-dependent (Hill, 2011; Garratt & Brooks, 2012; Weaver et al., 2017) and can for instance respond to parasite load and oxidative stress (Maan et al., 2006; Blount & McGraw, 2008; Mougeot et al., 2010; Alonso-Alvarez & Galván, 2011). This provides a basis for carotenoids to function as honest signals of quality (Svensson & Wong, 2011). In several species of birds and fish, the intensity of carotenoid-based coloration predicts competitive success (Bakker & Sevenster, 1983; Evans & Norris, 1996; Dijkstra et al., 2007; Murphy et al., 2009). Effects of color intensity on social interactions in *Tropheus* “Ikola” have not yet been tested, but are suggested by the flexible enhancement of color contrast and intensity to signal dominance, as described above. We hypothesized that bar width can contribute to the information content of the color signal. Low-quality individuals may be unable to muster sufficient amounts of carotenoids for the intense pigmentation of wide bars because of physiological limitations or costs associated with integumentary carotenoid deposition (Hill, 2011; Weaver et al., 2017). Since integumentary carotenoids are also present in the black-colored body regions of *Tropheus* “Ikola” (Ziegelbecker et al., 2018), we first ascertain in the present study that the yellow-colored integument contains a higher concentration of carotenoids than the adjacent, black-colored integument. Hence, if the allocation of carotenoids to the integumentary coloration of *Tropheus* “Ikola” is condition-dependent, the competitive advantage of wide-barred females could arise because (1) brightly colored, wide bars signal superior quality and have an intimidating effect on competitors, and (2) because the expression of wide bars is correlated with competitive traits such as increased boldness and aggressiveness during territorial competition. We used painted fish dummies to elicit territorial behavior from focal fish and tested for effects of the competitors’ color patterns (dummies and focal fish) on the behavior of male and female focal fish. Given that the competitive advantage of wide bars was only observed in female–female competition, we predicted that any effects would be sex-specific.

## Materials and methods

Wild-caught *Tropheus* sp. ‘black’ from Ikola (Tanzania, Africa) were purchased from an ornamental fish trader. Fish were housed in same-sex group tanks (*L* × *W* × *D*, 150 × 70 × 50 cm) of 25–40 individuals, except for periods prior to each of the different experiments, when fish were kept separated for at least 2 weeks in individual tanks (*L* × *W* × *D*, 60 × 35 × 30 cm). Standard length (SL) of fish was determined shortly before behavioral experiments and bar width was measured from photographs as described in Ziegelbecker et al. (2018) to calculate relative bar width (bar width/SL). All tanks were filtered with internal box filters and illuminated with an overhead white light on a 12:12 h light/dark cycle. Housing tanks were heated to 24–27°C, and temperature in experimental tanks was held constant at 26 ± 0.5°C. Fish were fed once a day with flake food (Spirulina Super Forte, Tropical). Behavioral experiments were carried out at University of Graz between August 2016 and September 2017, and the carotenoid analyses presented here were conducted in 2018. Fish were continuously watched by the researcher (AZ) while in experimental trials. Housing, handling and euthanasia of the fish was covered by permit number BMWFW-66.007/0004-WF/V/3b/2016 issued by the Federal Ministry of Science, Research and Economy of Austria. This study was carried out with the ethical approval of the ethics committee of the University of Graz (permit number GZ. 39/45/63 ex 2019/20).

### General statistical procedures

All statistical analyses were conducted in R, version 3.6.3 (R Core Team, 2019). In analyses using general linear (mixed) models, we checked for normality of residuals (Shapiro–Wilk tests; all *P* > 0.1) and for patterns in the distribution of residuals using the function ‘check_model’ in the R package ‘performance’ (Lüdecke et al., 2020). If necessary, data were transformed and influential outliers removed, as detailed below, to meet model assumptions. We were interested in whether any effects of the focal fish’ or the dummy fish’ color patterns were sex-specific. The interaction terms (bar width of focal fish : sex; dummy type : sex) were non-significant in all models, which could, however, reflect a lack of power in some of the datasets. To avoid the data-hungry tests for interactions (e.g. Wahlsten, 1991), we then calculated separate models for male and female focal fish, including only main effects as described below. When datasets included repeated measurements of individual fish, i.e. tests of fish with different types of stimuli, we included fish identity as a grouping factor in the linear models and estimated intra-individual correlation (repeatability *R*, adjusted for effects of fixed factors) using the function rptGaussian in the package rptR (Stoffel et al., 2017). Reported *P* values were not corrected for multiple testing (Althouse, 2016). Significance of *R* was tested using likelihood ratio tests.

Figures were drawn in R (R Core Team, 2019) and/or Inkscape 0.92.4 (Inkscape project members, 2019). Boxplots represent medians, the first and third quartiles, the range of data within 1.5 interquartile distances above and below the interquartile (whiskers), and individual outliers (open circles).

### Integumentary carotenoid concentration

Carotenoids were extracted from skin and scales of three different regions of the body: within the yellow bar, the black area anterior of the yellow bar and the black area posterior of the yellow bar (Fig. 1a). The protocol followed recommendations for carotenoid extraction from multicellular tissue (Saini & Keum, 2018), including the lyophilization of tissue to reduce the water content, the addition of antioxidant to the solvent, the application of ultrasound and the protection of the sample from UV light during all steps. The use of acetone as solvent followed previously published protocols for extraction from the skin or fins of various fish species (e.g., Wedekind et al., 1998; Ohkubo et al., 1999; Grether et al., 2001; Eckmann et al., 2017) including cichlids (Maan et al., 2004; Mattersdorfer, 2011). In total, 24 individuals (18 males and 6 females) were analyzed. Six of the males had died roughly a year before the analysis and had been stored at − 20°C. The other fish were euthanized with an overdose of MS-222 (2 g/l aquarium water) and dissected directly afterwards. Scales were removed from the skin samples and collected separately, and skin samples were carefully separated from underlying tissue. The sizes (cm^2^) of the skin samples were measured from photographs of the dissected pieces using ImageJ (version 1.50i, Schneider et al., 2012), and the fresh weight of each sample was recorded. Next, skin samples were cut into little pieces of 2 × 2 mm and freeze-dried under vacuum in the dark for 3 days. We also took photographs of the scales and measured the size of pigmented area of each scale, which corresponds to the region of the scale on the surface of the integument, i.e. not overlaid by other scales. Scales were also weighed and then freeze-dried under vacuum in the dark for 3 days.

Then, we determined the dry weight of the skin and scale samples, cooled them briefly in liquid nitrogen and ground them in a high frequency vibrating mill (30 Hz) for 3 min. Directly after grinding, samples were placed in 1.5 ml tubes and covered with 1 ml acetone mixed with antioxidant 2,6-di-*tert*-butyl-4-methylphenol (BHT, 1 g/l acetone). Extracts were vortexed, transferred into an ultrasonic water bath for 10 min and then centrifuged for 10 min in a high-speed centrifuge (~13,000 RCF, 4°C). The supernatant was transferred and the remaining pellet extracted a second time using the same procedure except for a longer incubation (overnight at 4°C). On the next day, the supernatant of the second extraction was added to the first extraction and samples were filled up to a volume of 2 ml (with acetone-BHT, 1 g/l). Pilot experiments showed that a third extraction would produce only negligible additional yield (4% increase to the first two extracts). Absorbance of each sample across wavelengths of 350 to 600 nm was measured using a Hitachi U3000 Spectrophotometer equipped with the program UV Visions 1.0 (Hitachi). Samples with a maximum absorbance greater than 0.9 were diluted 1:5 and those whose maximum absorbance was greater than 2 were diluted 1:10 in acetone-BHT (1 g/l) before repeating the measurement.

HPLC analysis of carotenoid extracts were conducted in the context of another study and revealed that the carotenoids in the integument of *Tropheus* “Ikola” are mainly xanthophylls (unpublished data, Ziegelbecker and Sefc). Therefore, carotenoid concentration was calculated from absorbance measures based on the extinction coefficient of 2550 for unknown xanthophylls (Bauernfeind, 1981, as suggested in Clotfelter et al., 2007; see Online Resource 1). Carotenoid concentrations were calculated per area (µg per cm^2^ of skin or pigmented scale area) and per tissue weight (dry weight and fresh weight of each sample). To determine the carotenoid concentration per cm^2^ body surface, we summed the concentrations of carotenoids per cm^2^ skin and per cm^2^ colored scale area for each of the three examined body regions of each fish. We then used a general linear mixed model (R packages lme4, Bates et al., 2015, and lmerTest, Kuznetsova et al., 2017) to test for differences in carotenoid concentrations between yellow and black body regions, including concentration (µg/cm^2^ integument; log transformed) as dependent variable, body region as predictor and the individual fish as random factor. This way, we compared carotenoid concentrations within individual fish independent of among-fish heterogeneity in body condition or other factors that could potentially affect carotenoid concentrations in the integument.

### Experiment 1: Behavioral responses of focal fish towards fish dummies and an arbitrary stimulus object

**Fig. 2:**
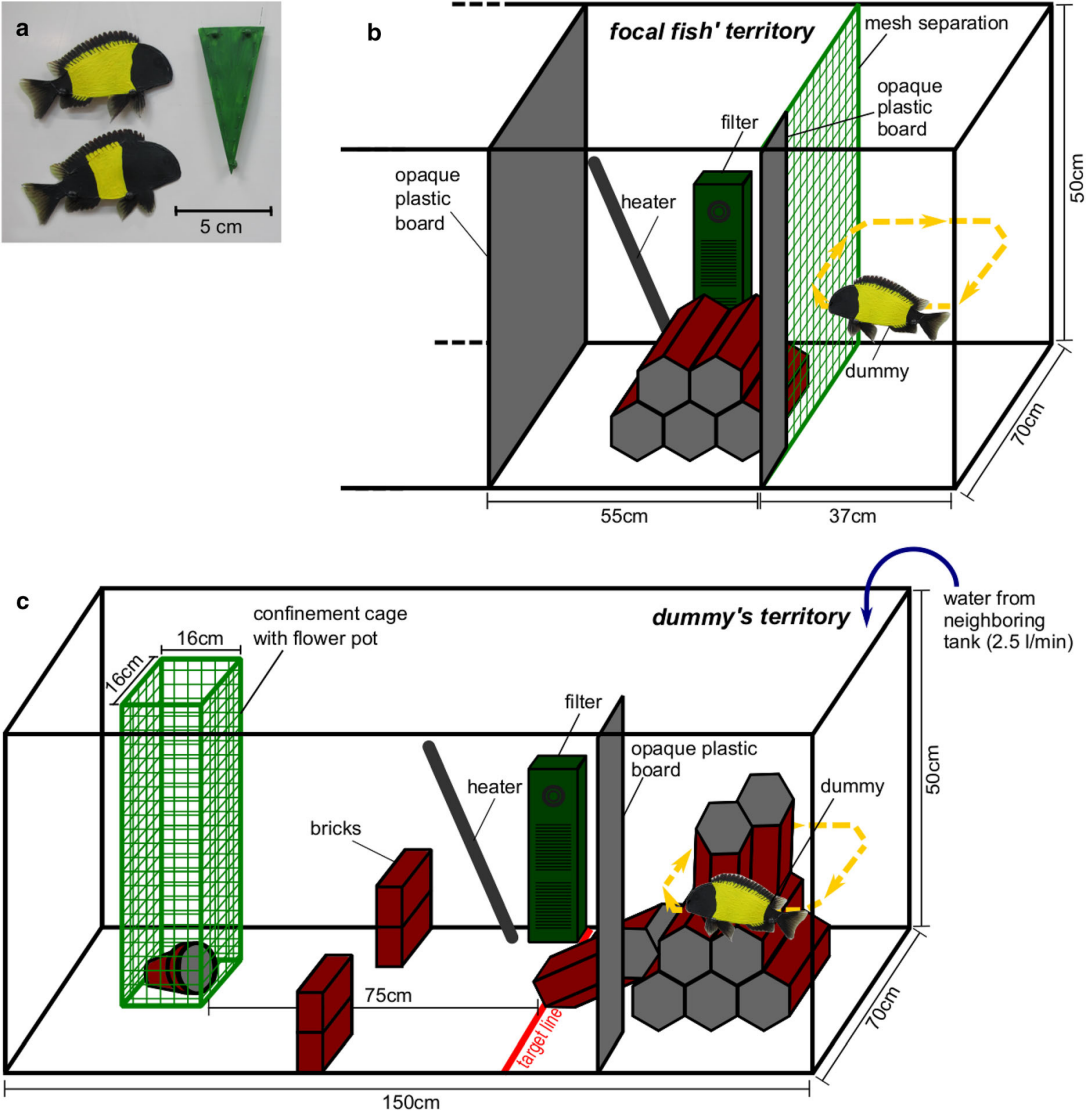
Stimulus objects used in the behavioral experiments (**a**) and setup of tanks in experiment 1 (**b**) and experiment 2 (**c**). Dummies in (**b**) and (**c**) are not drawn to scale

In this experiment, we studied behavioral responses of focal fish (18 males, 18 females) towards an arbitrary unfamiliar object (a thin and flat plastic triangle painted green, height 7.5 cm, tip pointing downwards; Fig. 2a), and a *Tropheus* “Ikola” dummy displaying a wide yellow bar and another dummy displaying a narrow yellow bar (Fig. 2a). The triangle was painted green, because this color is not displayed by fish in the natural habitat of *Tropheus* “Ikola” and therefore should not be associated with predation risk, territory competition or mating opportunity. Furthermore, the triangle was presented upright in order to minimize resemblance with fish shape. For fish dummies, a photo of a displaying individual (with fins erect) was printed on a plastic foil and repainted with yellow and black acrylic color to mimic the intensive coloration of a live fish demonstrating dominance. We prepared dummies of different body sizes (7.5–10.5 cm in 0.5 cm increments) such that dummies could be size-matched with focal fish (size differences between test fish and dummy, mean ± SD = 0.15 ± 0.20 cm). The widths of the bars of narrow-barred and wide-barred dummies were 21% and 48%, respectively, of their standard length. Hence, relative bar widths of dummies were designed to be somewhat narrower and wider, respectively, than those observed in the natural population, which ranged from 28 to 41% (*n* = 70 wild-caught fish of our stock). Sinkers were attached to the stimulus objects to keep them submersed during presentation. A fishing line was used to tie the objects to a motorized LEGO^®^Technic construction, which was placed on foam material (to absorb vibrations) above the tank. The objects (the triangle or one of the dummy types) were moved along a rectangular trajectory (27 × 55 cm) at a speed and (in case of the fish dummies) in a posture that mimicked patrolling *Tropheus*.

The experiment was conducted in a test arena within a large tank (150 × 70 × 50 cm, *L* × *W* × *D*) (see Fig. 2b). The arena was divided by a mesh partition (mesh size 13 mm) into two compartments, one of 55 × 70 × 50 cm for the focal fish, and the other of 37 × 70 × 50 cm for the presentation of the stimulus object. The compartment for the focal fish was equipped with a filter and a heater. Hollow clay bricks served as shelter and territory center and were placed directly adjacent to the mesh. Test fish were allowed 2–4 h (as needed) to settle and become territorial. Then, the stimulus was introduced and moved seven rounds (for a total of 4.5 min) along its trajectory next to the focal fish’ territory, mimicking a potential intruder. Each focal fish was exposed to each of the three stimulus objects in randomized order, with a 2-min break between trials. The focal fish were videotaped for analyses of their behavioral responses to the stimulus presentations. The videos did not show the stimulus object, and the video file names were manipulated in order to keep the experimenter blind to the type of stimulus and the identity of the focal fish during the scoring of the focal fish’ behavior. We counted the frequencies of the following types of behavior: charges towards the stimulus, lateral and frontal displays, in which the fish present themselves with erect fins either parallel or orthogonal to the stimulus’ trajectory, and swimming along with the stimulus. These types of behavior are part of the agonistic behavioral repertoire that is shared across cichlid species (Baerends & Baerends-van Roon, 1950) and also occur during agonistic interactions in the context of competition for territories among *Tropheus moorii* (Boulenger, 1898) individuals (Odreitz & Sefc, 2015). We addressed this set of behaviors as *charges and displays* in the remainder of the manuscript. Additionally, we observed flight (quick movement away from the stimulus) and freezing (lying motionless on the ground, no observable fin movement), which we scored as *fright behaviors*. Two females and one male showed no response to the stimulus objects and were excluded from the analysis.

We first tested whether focal fish differentiated between the fish dummies and the green triangle. To this aim, we summed all occurrences of charges and displays as well as all displays of fright behaviors performed by each fish towards each of the stimulus objects. We used generalized linear mixed models (GLMM) with a negative binomial error distribution (‘nbinom1’ in package glmmTMB, Brooks et al., 2017) to compare the total counts of charges and displays and the total counts of fright behaviors between presentations of the triangle, wide-barred and narrow-barred dummies. We included object type as fixed factor and fish identity as random factor, as each fish was exposed to each of the three stimuli. In this analysis, we combined male and female datasets (*n* = 33 fish).

Next, we focused on charges and displays against fish dummies. We first compared the summed counts of charges and displays against fish dummies (irrespective of dummy type) between male (*n* = 17) and female (*n* = 16) test fish, using a GLMM (binomial error distribution, ‘nbinom1’ in package glmmTMB, Brooks et al., 2017) with sex as fixed factor and fish identity as random factor. We then examined whether fish behaved differently towards wide-barred and narrow-barred dummies and whether the bar width of the focal fish affected their behavior. To this end, we first normalized the counts for frontal displays, lateral displays, swimming with dummy and charges towards dummy using their medians and interquartile ranges, and then summed the normalized values. This way we ensured that the variation of the different types of behaviors contributed equally to the summary value for charges and displays (sums of raw counts were driven by the high number of charges). The counts were processed separately for male and female datasets. We constructed general linear mixed models (R packages lme4, Bates et al., 2015, and lmerTest, Kuznetsova et al., 2017) including the summary values for charges and displays as dependent variable. As predictors, we included dummy type and relative bar width of the focal fish. Since each fish was presented with both types of dummies, we also included a fixed factor, which indicated whether the data point represented the first dummy presentation for this focal fish, and we included fish identity as random factor. The continuous predictor (relative bar width) was scaled and centered.

### Experiment 2: Exploration and territory intrusion by the focal fish

In this experiment, we examined the latency of focal fish to explore an unfamiliar but attractive region of the test tank. An experimental tank (150 × 70 × 50 cm, *L* × *W* × *D*) was furnished as shown in Fig. 2c. In one end of the tank, an arrangement of clay bricks provided an attractive structure (the target territory) for the rock-dwelling *Tropheus* (Hermann et al., 2015), but at the same time represented a complex, unfamiliar environment. At the opposite end of the tank, focal fish were confined to a wire cage (16 × 16 × 50 cm; mesh size 13 mm) which was equipped with a small flower pot for shelter. Bricks stacked to the left and right in front of the confinement cage restricted the possibilities for visual inspection of the remaining tank space by the focal fish during confinement. The walls of the experimental tank were coated with white plastic boards to avoid any visual disturbance from outside.

In the first part of the experiment, we ascertained that the fish were attracted to the target territory and examined whether relative bar width of the focal fish related to the time to intrude into the attractive, but unfamiliar part of the test tank. After 8 min of acclimation in the confinement cage, the test fish were released by carefully lifting the cage. The test fish were videotaped from above. Latency to explore the unfamiliar target territory was measured as time between lifting of the confinement cage and crossing of a defined line in front of the target territory by the test fish (the *target line* in Fig. 2c). The motor of the construction, which was going to move the dummies in the second part of the experiment, was also turned on here while the focal fish was confined to the cage, although no dummies were presented. Out of 66 tested fish, only 4 did not enter the target area within 5 min. The remaining trials were included in the analysis, but one further male was dropped after it was identified as influential outlier during model checking, resulting in a sample size of 34 females and 27 males in the final models. We first analyzed males and females separately, and used general linear models (R packages lme4, Bates et al., 2015, and lmerTest, Kuznetsova et al., 2017) to test whether the relative bar width of the test fish influenced its latency time. Latency times were square root transformed, and the continuous predictor (relative bar width) was scaled and centered. Male and female datasets were then combined and examined for sex differences in latency time (square root transformed) in a general linear model with sex and relative bar width (scaled and centered) as fixed factors.

The second part of the experiment was carried out several months after the first part had been completed, using a subset of the fish that had performed in the first part of the experiment. Here, we tested whether the color pattern of the ‘owner’ (represented by wide- and narrow-barred dummies) of the target territory affected the latency to intrusion. To this aim, a size-matched dummy (size differences between test fish and dummy, mean ± SD = 0.14 ± 0.23 cm) displaying either a narrow or a wide bar was moved around the brick structure in the target territory using the construction described above (see experiment 1, Fig. 2c). The fish dummy was only presented during the 6-min acclimation period while the test fish was confined to its cage. The test fish could see the target territory and the patrolling dummy. At the end of the acclimation period and when the dummy was hidden from the test fish behind an opaque plastic board attached to one side of the arena, the device was turned off and the dummy was removed before the test fish was released from the confinement cage. Time between release from the confinement cage and crossing of the target line was again determined based on video recordings. Since *Tropheus* “Ikola” are sexually monomorphic in color pattern and body size, the test fish could interpret the dummies as same sex competitors or as potential mating partners. Sexually motivated behavior would have interfered with the aim of the experiment, which was concerned with competition for territories. Therefore, we attempted to provide chemical cues to inform the test fish about the ‘sex’ of the dummy. During social interactions, cichlid fish release chemical signals, including sex hormones, into the water (Hirschenhauser et al., 2004; Keller-Costa et al., 2015), and exposure to male- or female-conditioned water for instance triggers different responses from males of *Astatotilapia burtoni* (Günther, 1894) (Crapon de Caprona, 1980). We, therefore, introduced water from an adjacent tank (tank size 450 l), which housed eight adult fish of the same sex as the test fish, into the target territory area of the test tank during the presentation of the dummy fish (inflow 2.5 l/min). Latency data obtained in a pilot experiment (*n* = 34 females and 25 males), which was conducted as described here but without the addition of conditioned water, are referred to in the discussion for comparison with the experiment including inflow of conditioned water.

Each focal fish was tested in two sessions, which took place in the morning and in the afternoon (5 h apart) of the same day, once against the wide-barred and once against the narrow-barred dummy. We randomly assigned dummy type for use in the first and in the second session. After exclusion of fish which did not enter the target territory (5 males and 1 female), we had 7 females and 9 males starting with the narrow-barred dummies and 8 females and 6 males starting with the wide-barred dummy (total sample size in analysis = 15 males and 15 females). The sex difference in latency time was examined in a general linear model with log transformed latency time as dependent variable, sex as fixed factor and fish identity as a random factor. Effects of color pattern on latency time were then analyzed separately for males and females, using general linear mixed models (R packages lme4, Bates et al., 2015, and lmerTest, Kuznetsova et al., 2017), with latency time (log transformed) as dependent variable and dummy type and relative bar width of focal fish (scaled and centered) as predictors. We also included a fixed factor which indicated whether the session had taken place in the morning or in the afternoon, and fish identity as a random factor. Session (morning vs. afternoon) had no significant effect on latency times and was dropped from the final models.

## Results

### Carotenoid concentration in yellow and black integument

Carotenoid concentrations were measured in the skin and scale samples taken from yellow and black body regions (Table 1). Concentrations per cm^2^ skin and colored scale area were then added to obtain the total carotenoid content per cm^2^ body surface. Across 24 fish, the carotenoid concentration amounted to 6.78 ± 1.76 µg/cm^2^ (mean ± SD) in the yellow region, and was significantly lower in both the anterior black region (2.77 ± 1.19 µg/cm^2^; est. = − 0.97, *t* = − 13.19, *P* < 0.001) and the posterior black region (2.03 ± 0.85 µg/cm^2^; est. = − 1.30, *t* = − 17.23, *P* < 0.001). The difference in carotenoid concentration between the anterior and the posterior black region was also significant (est. = − 0.34, *t* = − 4.45, *P* < 0.001). Because of the low sample size for females (*n* = 6), we did not test for sex-specific differences in the distribution of carotenoids, but the mean values of carotenoid concentrations in the three body regions show congruent patterns for males and females (mean ± SD µg/cm^2^ in males, anterior black = 2.68 ± 1.34, yellow = 6.36 ± 1.70, posterior black = 1.92 ± 0.94; females, anterior black = 3.01 ± 0.56, yellow = 8.06 ± 1.36, posterior black = 2.33 ± 0.49; see also Fig. 1b).

**Table 1:** Carotenoid concentrations in skin and scale samples from the three analyzed body regions (see Fig. 1a)

Body region/tissue	Dry weight (µg/g)		Fresh weight (µg/g0)		Body surface (µg/cm^2^)	
	Mean	SD	Mean	SD	Mean	SD
Anterior black region						
Skin	583.8	257.65	134.9	71.33	2.2	1.07
Scales	16.0	6.91	11.3	5.73	0.5	0.21
Yellow bar region						
Skin	1021.8	408.18	217.1	68.46	5.5	1.54
Scales	36.0	15.04	24.6	12.23	1.3	0.49
Posterior black region						
Skin	368.3	184.20	97.0	57.76	1.7	0.78
Scales	14.2	6.60	10.2	5.45	0.3	0.12

Concentrations were calculated in relation to fresh and dry weight of the samples and to area of body surface. Means and standard deviations (SD) across 24 *Tropheus* “Ikola” are given

Controlling for body size, the area of the yellow bar differed between individual fish by up to 2.8 cm^2^ per body side (*n* = 70 fish), with wider bars extending farther towards the tail fin than narrower ones. Based on these results, we estimate that the absolute difference in integumentary carotenoid content between narrow- and wide-barred individuals can reach up to 25 µg (4.75 µg/cm^2^ difference between mean concentrations in yellow and posterior black body region and up to 2.8 cm^2^ difference in yellow area size between individuals at each side of the body; note that this estimate does not include the yellow coloration in the proximal part of the dorsal fin).

### Experiment 1: Focal fish behavior towards objects and fish dummies

**Fig. 3:**
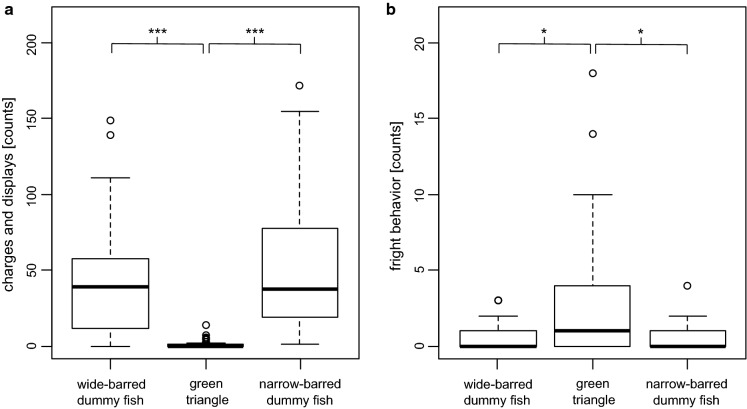
Behavior of focal fish (*n* = 33) towards stimulus objects (wide-barred dummy fish, narrow-barred dummy fish, green triangle). The boxplots show counts of **a** charges and displays and **b** fright behavior within 4.5 min of stimulus presentation. *** and * indicate *P* < 0.001 and 0.01 < *P* < 0.05, respectively

We first tested whether focal fish (irrespective of their sex) differentiated between the fish dummies and an arbitrary foreign object (green triangle). Focal fish performed significantly more charges and displays towards fish dummies than towards the triangle (Fig. 3a; 0–172 with median = 38.5 behaviors counted per session against dummy fish; 0–14 with median = 0.0 per session in triangle presentations; *n* = 33; est. = 2.55, *P* < 0.001 and est. = 2.72, *P* < 0.001 for triangle versus wide- and narrow-barred dummy, respectively; separate analyses of males and females gave nearly identical results, data not shown). On the contrary, fish dummies elicited significantly fewer fright behaviors than triangles did (Fig. 3b; 0–4 with median = 0.0 behaviors counted per session with dummy fish presentation; 0–18 with median = 1 per session in triangle presentations; *n* = 33; est. = − 0.86, *P* = 0.024 and est. = − 0.90, *P* = 0.023 for triangle versus wide- and narrow-barred dummy, respectively).

In trials with fish dummies, male test fish performed somewhat more charges and displays per session (*n* = 17; 5–172 with median = 48 behaviors counted per session against dummy fish) than female test fish (*n* = 16; 0–111 with median = 27.5; est. = 0.48, *t* = 1.95, *P* = 0.051). Separated by dummy type, female test fish performed 1–108 (median = 27.5) and 0–111 (median = 31) charges and displays per session against narrow-barred dummies and wide-barred dummies, respectively. Males performed 5–172 (median = 49) charges and displays per session against narrow-barred dummies and 6–149 (median = 48) per session against wide-barred dummies.

We then analyzed the sums of the normalized counts of charges and display behaviors towards fish dummies separately in male and female focal fish. In males, fewer charges and displays were elicited by wide-barred dummies than by narrow-barred dummies (est. = − 1.10, *t* = − 2.30, *P* = 0.037), and males (but not females) decreased the number of charges and displays between the first and the second dummy presentation (est. = − 2.08, *t* = − 4.34, *P* < 0.001). We detected no effect of the relative bar width of the focal fish (Table 2). Repeatability within individual fish was significant in both sexes (females, *R* = 0.56, *D* = 6.01, *P* = 0.007; males, *R* = 0.58, *D* = 7.01, *P* = 0.004).

**Table 2:** General linear (mixed) models testing for effects of dummy type and the relative bar width of focal fish on the behavior of male and female focal fish

(a) Experiment 1: Charges and displays^a^								
	Females (*n* = 16)				Males (*n* = 17)			
	*β*	SE	*t*	*P*	*β*	SE	*t*	*P*
Dummy type (wide-barred)	− 0.530	0.671	− 0.789	0.443	− 1.098	0.478	− 2.295	**0.037**
Relative bar width^b^	0.044	0.642	0.069	0.946	0.076	0.471	0.161	0.874
First dummy presentation (yes)	0.778	0.671	1.158	0.266	2.078	0.478	4.343	**0.0006**

(b) Experiment 2: Latency time to exploration^c^								
	Females (*n* = 34)				Males (*n* = 27)			
Relative bar width^b^	− 1.590	0.766	− 2.076	**0.046**	− 1.057	0.820	− 1.289	0.209

(c) Experiment 2: Latency time to intrusion^d^								
	Females (*n* = 15)				Males (*n* = 15)			
Dummy type (wide-barred)	− 0.109	0.224	− 0.488	0.633	0.279	0.275	1.014	0.328
Relative bar width^b^	0.393	0.255	1.542	0.147	− 0.351	0.258	− 1.359	0.197

(a) Charges and displays of focal fish against wide-barred and narrow-barred dummies, (b) latency time to exploration of an unfamiliar area of the tank (without dummy presentation), (c) latency time to intrude into a territory guarded by a wide-barred or a narrow-barred dummy. Effect estimates (*β*), standard error (SE), t-value and *P* value are shown for each of the fixed factors included in each respective model. Significant *P* values are indicated in bold

^a^The counts for each behavior (frontal displays, lateral displays, swimming with dummy and charges towards dummy) were normalized using their medians and interquartile ranges, and then summed up and collectively referred to as *charges and displays*

^b^Relative bar width was scaled and centered

^c^Square-root transformed

^d^Log transformed

### Experiment 2: Time to exploration and intrusion by focal fish

**Fig. 4:**
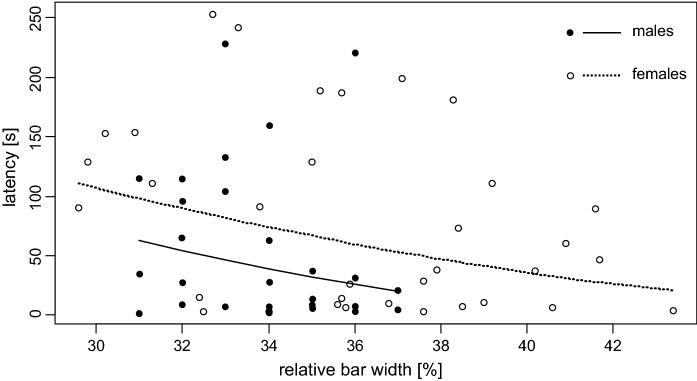
Latency to explore an unfamiliar tank space in relation to the color pattern (relative bar width) of the focal fish (27 males, 34 females; full circles = males, empty circles = females). The solid and the dashed line represent the model predictions for males and females, respectively

We asked whether the propensity to intrude into foreign territories (represented by latency time) depended on traits of the focal fish and differed when territories were held by a narrow-barred or by a wide-barred dummy. When no dummy was present in the target territory, latency times of focal fish ranged from 2 to 253 s (mean ± SD for females: 79.68 ± 76.97, *n* = 34; for males: 57.45 ± 64.30, *n* = 27). Latency time decreased with relative bar width in females (Fig. 4; est. = − 1.59, *t* = − 2.08, *P* = 0.046) but not significantly so in males (Fig. 4, Table 2). With data from male and female fish pooled into one dataset (*n* = 61 fish), latency time again decreased with relative bar width (est. = − 1.46, *t* = − 2.47, *P* = 0.017; the interaction term between sex and bar width was not significant) and latency times of males were significantly shorter than those of females (est. = − 2.41, *t* = − 2.04, *P* = 0.046; Fig. 4).

When fish dummies were displayed within the target territories, latency times ranged from 3 to 169 s (mean ± SD = 38.0 ± 36.7 s in males and 54.4 ± 45.5 s in females). The difference between males and females was not significant (est. − 0.45, *t* = − 1.22, *P* = 0.23). Latency time did not co-vary with dummy type or bar width in the male and female groups (Table 2). Repeatability within individual fish was significant in both sexes (females, *R* = 0.67, *D* = 8.12, *P* = 0.002; males, *R* = 0.55, *D* = 4.75, *P* = 0.015).

## Discussion

Associations between body coloration and success in contest competition can be due to intimidating effects of coloration on opponents (Evans & Norris, 1996; Baube, 1997; Dijkstra et al., 2005; Healey et al., 2007; Pryke, 2009). To test this, it is necessary to expose test subjects to a variation in the color pattern of interest. Experimental manipulation of body coloration is difficult to apply in fish (Rowland, 1999; for examples see Theis et al., 2012; Bachmann et al., 2017) and fish models and computer animations have turned out as useful alternatives to study effects of trait variation on social and sexual interactions (Rowland et al., 1995a, b; Lehtonen, 2014; Kohda et al., 2015; Lehtonen et al., 2015; Balzarini et al., 2017). Moreover, presentations of dummies and video playbacks allow investigators to keep other variables, for instance the behavior of the stimulus, constant across the experiment. The use of dummies to study fish behavior is facilitated by the fact that some fish display social and sexual behaviors even to unrealistic stimuli (Manning & Dawkins, 2012). In our study, we ascertained that the test fish did not perceive the dummies in the same way as an arbitrary foreign object. Territorial test fish responded to introduced dummies with the same types of aggressive behavior (displays and charges), which *Tropheus* (and other cichlid fish) also express towards live competitors in contest competition (Baerends & Baerends-van Roon, 1950; Odreitz & Sefc, 2015), whereas the non-fish object elicited almost no such behaviors.

Although test fish of both sexes clearly distinguished between the fish dummies and the non-fish object, only the male test fish responded to dummy type and performed fewer aggressive behaviors against wide-barred dummies than against narrow-barred dummies. In males, the number of aggressive behaviors further decreased between first and second dummy presentations. This may represent habituation to the experimental stimulus (i.e. be an experimental artifact), but may alternatively reflect a characteristic of male territoriality, if males reduce the frequency of charges and displays when they become accustomed to the presence of territorial neighbors. In the second experiment, dummy type did not affect the time which test fish took to enter a sector of the experimental tank that had been presented as occupied by either a narrow-barred or a wide-barred dummy. Overall, the effects of dummy type in our experiments were rather weak in comparison to several other studies, which examined response times and aggression rates to presentations of dummies, video images or manipulated live stimuli. For instance, male lizards of the species *Intellagama lesueurii* (Gray, 1831) responded to models with red ventral coloration with longer latency and fewer displays and approaches compared to brown colored models (Baird et al., 2013), and male stickleback fish performed more attacks against moderately colored than against brightly colored video images (Rowland et al., 1995a). In experiments using live stimuli, juvenile Gouldian finches were slower to initiate contests with males whose heads were painted red than with black- or blue-painted males (Pryke, 2009), and male sticklebacks were quicker to start aggressive behaviors when the red belly color of their opponents was masked by the light environment than when it was visible to them (Baube, 1997).

The weak responses of our test fish to the (dummy) competitors’ bar width suggest that wide bars are not perceived as particularly intimidating, but may also reflect shortcomings of the dummy presentations. Zebra finch females, for instance, showed sexual behavior in response to video images of males, but their mate preferences differed significantly between presentations of live males and presentations of videos of the same males (Swaddle et al., 2006). *Tropheus* employ physiological changes of body color intensity as a means of communication (Yanagisawa & Nishida, 1991; Sturmbauer & Dallinger, 1995; Ziegelbecker et al., 2018), and the effect of bar width may, in live fish, be enhanced by intensity signaling. Possibly, the lack of species-specific behavior and color changes in the stimuli interfered with the differentiation between narrow- and wide-barred dummies by the focal fish. The intrusion latency of focal fish in reaction to distantly displayed dummies (experiment 2) would be expected to be less affected by this than their close-range aggressive behavior in experiment 1. In contrast, aggressive behavior of males was attuned to dummy type, while intrusion latencies did not vary with dummy type. Finally, the high repeatability of behaviors within individuals raises the possibility of intrinsically limited flexibility to react to external stimuli. It is, however, questionable whether such inflexible behavioral patterns would be compatible with the demands of the social environment experienced by the fish (Yanagisawa & Nishida, 1991; Sturmbauer & Dallinger, 1995), and the fish could still be expected to modulate their behavioral responses relative to their individual intrinsic baselines.

Since the color pattern of *Tropheus* “Ikola” is sexually monomorphic, the fish dummies were ambiguous with respect to which sex they represented and may have elicited either socially or sexually motivated behaviors in the focal fish. We tried to circumvent this problem in the latency experiment by releasing water conditioned by a group of same-sex individuals into the test tank. Cichlid fish are known to use chemical communication during reproduction, brood care and contest competition and are for instance able to infer the reproductive stage and dominance status of conspecifics from chemical cues released into the water (Keller-Costa et al., 2015) and to differentiate between male- and female-conditioned water (Crapon de Caprona, 1980). The addition of conditioned water indeed changed the behavior of the focal fish compared to an identical pilot experiment, but without the inflow of conditioned water. In the pilot experiment, average female latencies were twice as long as average male latencies (mean ± SD = 46 ± 44 s versus 21 ± 36 s, *t* = 3.43, *P* < 0.001 with *n* = 34 females and 25 males) and coefficients of variation were substantially larger in males (1.72) than in females (0.94). In comparison, in the experiment which presented the dummy fish with the treated water, the difference between average male and female latency times was not significant and coefficients of variation were more similar between the sexes (0.80 in females, 0.97 in males). This suggests that male focal fish, in particular, were affected by uncertainties about the sex represented by the fish dummies in the pilot experiment, and a portion of them may have moved more quickly into the dummy’s territory to pursue putative mating opportunities.

We also examined whether the dominance of wide-barred females in the experiment by Ziegelbecker et al. (2018) could be explained by correlated behavioral traits (Schweitzer et al., 2015; Dijkstra et al., 2017; Bohórquez-Alonso et al., 2018). Individual levels of aggressiveness (e.g. Riebli et al., 2011; Tinghitella et al., 2018) and boldness (Ward et al., 2004; Dahlbom et al., 2011) can predict competitive success, and if coincident with the expression of wide bars, could therefore underlie the correlation between bar width and dominance. In contrast to these studies, we detected no relationship between the focal fish’ bar width and rates of aggression towards fish dummies. However, we found that fish with wider bars (relative to their body size) were quicker to explore an unfamiliar tank area. Although the effect of bar width on latency was congruent in male and female test groups (Fig. 4), the relationship was significant only in the female group (and when the sexes were pooled for analysis), perhaps due to the narrower variance in the bar width among the tested males (which was an experimental artefact due to availability of test fish). Notably, the effect of the focal fish’ bar width on latency time was detected only in the trials without dummy fish presentations.

Males were quicker to move into the unfamiliar tank area and performed charges and displays against dummy intruders at significantly higher frequencies than females. Differences in the intensity of territory defense between the sexes may be due to differences in resource value for males and females (Draud et al., 2004). In *Tropheus*, both sexes derive food and shelter from their individual territories (Yanagisawa & Nishida, 1991), but asymmetries in resource value can arise because males additionally depend on territories for their mating success (Hermann et al., 2015). In contrast to our study, a previous experiment in another *Tropheus* species, *T. moorii* (population “Moliro”), detected no differences between males and females regarding the types and rates of aggressive behavior during contest competition and suggested that inter-sexual competition for territories selected for sex-independent aggressive behavior (Odreitz & Sefc, 2015). However, in that experiment, individuals competed for the acquisition of new territories, while the present experiment was concerned with territory defense. This suggests that the degree of sexual monomorphism in contest behavior may be context-dependent or vary between *Tropheus* species and populations.

Our pigment analyses demonstrated that the allocation of carotenoids to body coloration increases with bar width (i.e., the area of the yellow bar), as the carotenoid concentration per unit body surface was about three times as high in the yellow integument compared to the adjacent black integument. However, the carotenoid concentration in the integument of the black-colored body regions was surprisingly high as well. Comparisons of carotenoid concentrations in animal tissue across studies are hampered by the use of different extraction and detection protocols, but we note that the amount of carotenoids measured in the black-colored integument of *Tropheus* “Ikola” (Table 1) was similar to that reported for the red-colored belly patch of male sticklebacks (150 µg/g fresh weight; Pike et al., 2011). However, the integumentary carotenoids in the black body regions of *Tropheus* “Ikola” are not displayed in the color pattern. From the perspective of carotenoids as costly ornaments that have to be diverted from physiological functions, this seems a wasted expenditure. Integumentary carotenoids have also been detected outside of red or yellow color patches in other color pattern variants of *Tropheus* (Mattersdorfer et al., 2012), and further work is needed to identify the function of these non-displayed pigments. Skin carotenoids might for instance play a role in photoprotection in the sun-lit shallow water inhabited by *Tropheus* (Blount & McGraw, 2008). Moreover, carotenoids may not be a limiting resource for *Tropheus*, which ingest carotenoid-rich cyanobacteria and chlorophytes by browsing epilithic algae in the rocky littoral (O’Reilly, 2001; Hata et al., 2014). Hence, an increase of yellow area in the color pattern of *Tropheus* “Ikola” may be associated with negligible allocation costs.

The experiments presented in this study suggested an association between the width of the yellow bar and the readiness of test fish to explore a novel environment. Furthermore, wide bars on a dummy competitor inhibited aggressive acts of male test fish compared to presentations of narrow-barred dummies. While our results suggest that intimidation by wide bars as well as correlations between bar width and explorative behavior may contribute to mediating success in territorial interactions in *Tropheus* “Ikola”, we did not detect the sex-specific effects which were predicted by the competitive advantage of wide bars in female–female, but not male–male contest competition (Ziegelbecker et al., 2018). The high concentration of integumentary carotenoids in the black-colored body regions suggests that carotenoids may not be a particularly limited resource for *Tropheus* “Ikola”, which would relax any condition-dependent constraints on bar width in this fish.

## Electronic supplementary material

Below is the link to the electronic supplementary material.

Supplementary material 1 (PDF 614 kb)

Supplementary material 2 (XLSX 83 kb)

## Data Availability

The datasets generated and analyzed during the current study are available in Online Resource 2.
