# Supplementary material for: Wasteful carotenoid coloration and its effects on territorial behavior in a cichlid fish
Source: Hydrobiologia. 2020 Aug 18;848(16):3683–98. doi: 10.1007/s10750-020-04354-3 (PMC8549929; doi:10.1007/s10750-020-04354-3)
Supplement: Supplementary file 1 — Supplementary material 1 (PDF 614 kb) [file 10750_2020_4354_MOESM1_ESM.pdf]

Online Resource 1 Spectrophotometry of carotenoid extracts and calculations of carotenoid concentrations

Wasteful carotenoid coloration and its effects on territorial behavior in a cichlid fish

Angelika Ziegelbecker, Klaus Remele, Hartwig W. Pfeifhofer, Kristina M. Sefc

Hydrobiologia

[1]University of Graz, Institute of Biology, Universitätsplatz 2, 8010 Graz, Austria
[2]University of Graz, Institute of Biology, Schubertstraße 51, 8010 Graz, Austria

[3]corresponding author: kristina.sefc@uni-graz.at, +43-(0)316-3805601, +43-(0)316-3809875

Absorbance spectra of carotenoid extracts

Absorbance spectra gained by spectrophotometry (Hitachi U3000) from analysis of carotenoid extracts from skin and scale samples were similar between tissue type and all three analyzed body regions. They had three peaks with maxima at approximately 418, 441 and 470 nm and a valley at approximately 458 nm (example given in Fig. S1). In samples with absorbance > 0.1, the average III/II value across samples was 86 ± 3. Spectra of samples with absorbance < 0.1 typically lacked pronounced peaks. Absorbance values for each tissue sample are given in online resource 3 (Datasets generated and analyzed during the current study)

Fig. S1 Absorbance spectrum of a carotenoid extract from *Tropheus* sp. 'black' skin (yellow bar tissue) gained by a wavelength scan of 350 – 600 nm

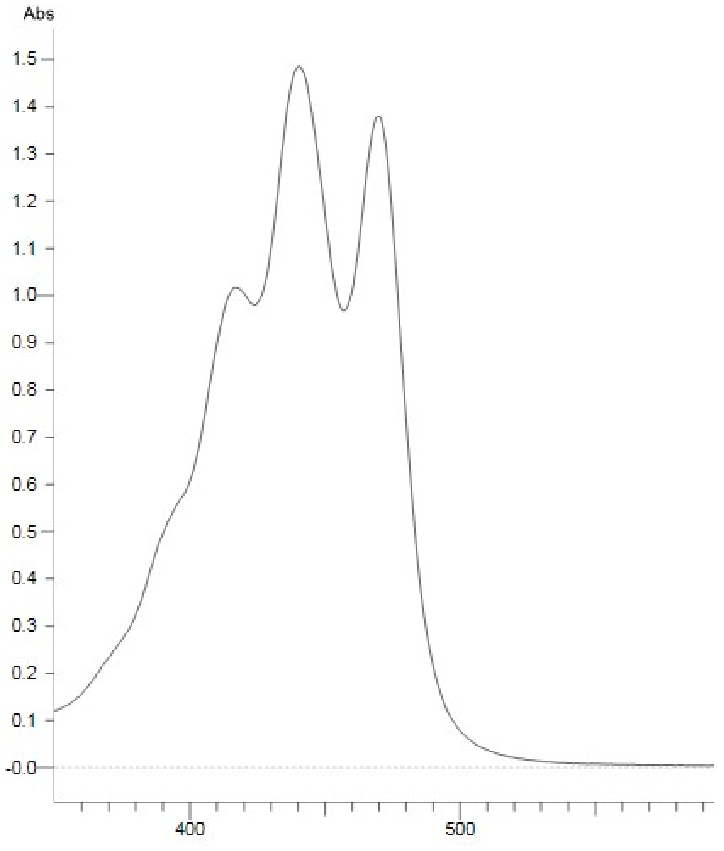

Calculation of carotenoid concentration

We calculated carotenoid concentrations in µg per cm² body surface, as well as in µg per g dry weight or fresh weight of the sample using the following formulas:

$A_{[Au]}$ x $V_{[ml]}$ x 10 x d x 1000 / ($E_{[10mg/ml]}$) x $w_{[g]}$) = carotenoid concentration per weight in sample $_{[µg/g]}$

$A_{[Au]}$ x $V_{[ml]}$ x 10 x d x 1000 / ($E_{[10mg/ml]}$) x $a_{[cm2]}$) = carotenoid concentration per area in sample $_{[µg/cm2]}$

A: maximum absorbance of the sample at the 441 nm peak of the spectrum
V: extraction volume
d: the dilution factor (e.g., d = 1 for samples not diluted and d = 5 for samples diluted 1:5)
E: extinction coefficient $A_{1\%1cm}$, which is "defined as the theoretical absorbance of a 1% solution, i.e. 1 g in 100 ml, in a cell of 1 cm pathlength" (Britton et al., 1995). Here, we used 2550 as the extinction coefficient for unknown xanthophylls (Bauernfeind, 1981), as suggested by Clotfelter et al. (2007).
w: weight of the extracted sample (fresh weight or dry weight)
a: area of body surface dissected for sample preparation

References

Bauernfeind, C. J., 1981. Carotenoids as colorants and vitamin A precursors. Academic Press, New York.

Britton, G., S. Liaaen-Jensen & H. Pfander, 1995. Carotenoids. Volume 1B, Spectroscopy. Basel.

Clotfelter, E. D., D. R. Ardia & K. J. McGraw, 2007. Red fish, blue fish: Trade-offs between pigmentation and immunity in Betta splendens. Behavioral Ecology 18: 1139–1145.
